# Locoregional Therapies for Hepatobiliary Tumors: Contemporary Strategies and Novel Applications

**DOI:** 10.3390/cancers16071271

**Published:** 2024-03-25

**Authors:** Andrei M. Jipa, Mina S. Makary

**Affiliations:** Division of Vascular and Interventional Radiology, Department of Radiology, The Ohio State University Medical Center, Columbus, OH 43210, USA; andrei.jipa@osumc.edu

**Keywords:** chemoembolization, radioembolization, thermal ablation, hepatocellular carcinoma, cholangiocarcinoma

## Abstract

**Simple Summary:**

Tumors originating in the liver and biliary system remain a significant health concern globally. The majority are cancers originating in liver cells known as hepatocellular carcinomas. Chronic liver disease including cirrhosis is a major risk factor for this type of cancer. For many patients, surgery is not an option due to the size of the tumor or the severity of liver disease. Established alternatives to surgery include chemoembolization, radioembolization, and ablation. These can be offered to a wide range of patients and produce similar outcomes to surgery for small tumors.

**Abstract:**

A large majority of primary hepatobiliary tumors are hepatocellular carcinomas (HCC), with the remainer being cholangiocarcinoma. While surgical resection remains the gold standard treatment for hepatobiliary tumors, relatively few patients are operative candidates, and systemic treatments have limited effectiveness. Locoregional therapies offer significant promise in the management of HCC. Ablation and radioembolization may offer similar outcomes to surgery for early-stage hepatocellular carcinoma while radioembolization and chemoembolization are valuable in the management of advanced disease. There is significantly less evidence for the role of locoregional therapy in the treatment of cholangiocarcinoma, although it appears to be well tolerated.

## 1. Hepatobiliary Tumors

Primary tumors of the liver and bile ducts are relatively common and may be benign or malignant. Benign neoplasms such as focal nodular hyperplasia (FNH), hepatic adenoma, or biliary adenoma/cystadenoma are typically managed with surgical resection or observation and will not be further discussed. Primary malignant neoplasms come in various histologic types, although all except hepatocellular carcinoma and cholangiocarcinoma are rare.

### 1.1. Hepatocellular Carcinoma

Hepatocellular carcinoma (HCC), also known as hepatoma, is the most common liver tumor and originates in hepatocytes. The primary risk factor is hepatic cirrhosis. HCC can be considered a complication of cirrhosis; therefore, it is commonly associated with major causes of cirrhosis including alcohol use, metabolic steatohepatitis/steatosis (MASH), hepatitis C infection, and chronic hepatitis B infection. In the context of cirrhosis, HCC carcinogenesis is felt to progress as a stepwise process from a regenerative cirrhotic nodule to dysplastic nodule to small HCC. Afterwards, HCC will either grow as a mass or spread diffusely within the liver. Remote or regional metastases are uncommon, although this also reflects the poor survival of patients as liver function declines due to intrahepatic tumor spread and progression of underlying liver disease.

Development of HCC outside the setting of cirrhosis is uncommon. Chronic hepatitis B without cirrhosis is also a known risk factor for HCC. This is a major driver of HCC in endemic areas such as South Asia and East Asia. Aflatoxin exposure has also been proposed as a potential risk factor for HCC in Asia. Fibrolamellar HCC is a rare but well-recognized subtype that is typically not seen in cirrhosis, usually presenting as a large liver mass in the second or third decade of life.

HCC is a biologically and morphologically diverse tumor that may be present as a unifocal mass, multifocal mass, or diffuse malignant infiltration of the liver. Infiltrative growth poses a serious challenge to non-invasive diagnosis and is associated with a poor prognosis. Histologically, HCC can overlap with adenocarcinoma, which is sometimes referred to as a “mixed” intrahepatic HCC and cholangiocarcinoma [1,2,3].

Staging of HCC is primarily based on statistical analysis of survival rates in patients with cirrhosis. The most used method is the Barcelona Clinic Liver Cancer (BCLC) staging system. BCLC divides HCC into early stage (A), intermediate-stage (B), advanced-stage (C) and terminal disease (D). Early-stage disease is defined as up to three lesions 3 cm or less in maximum diameter in the setting of compensated cirrhosis (Child class A or B) with preserved liver function and good performance status. A separate sub-classification of “very early stage” (0) disease is applied for a single lesion less than 2 cm in size. Intermediate-stage disease refers to compensated cirrhosis with more than 3 lesions or lesions greater than 3 cm in size. Tumor thrombus in the portal vein, extrahepatic spread, or mildly impaired performance status is classified as advanced-stage disease. The presence of decompensated cirrhosis (Child class C) or poor performance status defines terminal disease [2,4].

Surgical resection is considered the gold standard treatment for HCC and historically was the only treatment option with curative intent. Resection remains the mainstay of HCC management in patients without cirrhosis. Surgical risk is often prohibitive; however, due to underlying cirrhosis. Liver transplantation is also an established treatment for early-stage HCC within the Milan criteria which also manages the underlying cirrhosis. Systemic treatment is offered as an adjunct or palliative measure. Locoregional therapies therefore play two major roles in the management of HCC: (1) definitive treatment of localized HCC in patients who are high-risk candidates and (2) adjunctive treatment to downstage patients so they may be offered transplant, resection, or curative-intent locoregional therapy [2,4,5].

### 1.2. Cholangiocarcinoma

Cholangiocarcinoma refers to primary malignant tumors of the bile ducts and is the second most common primary malignant hepatobiliary tumor. These tumors are almost always adenocarcinomas, although biliary squamous cell carcinoma has been rarely reported. It is primarily a sporadic tumor seen in middle-aged and older adults. A minority of cases exist in the setting of established risk factors including primary sclerosing cholangitis (PSC), congenital biliary abnormalities such as choledochal cysts or Caroli disease, chronic liver disease including cirrhosis, recurrent pyogenic cholangitis, and liver fluke infections—the common agent being the presence of chronic biliary inflammation. PSC and cirrhosis are the most encountered risk factors in the Western World, whereas parasitic infections and recurrent cholangitis are dominant in Asia. PSC is strongly associated with inflammatory bowel disease (IBD), especially ulcerative colitis (UC), whereas recurrent pyogenic cholangitis is associated with intrahepatic choledocholithiasis and liver fluke infection.

The traditional classification of cholangiocarcinoma by location of origin is intrahepatic (proximal), perihilar (Klatskin tumor), and extrahepatic (distal). This distinction is based on surgical resection options. Intrahepatic tumors may be managed with partial hepatectomy while a Whipple resection may be possible for distal tumors. Perihilar tumors are typically not amenable to resection. Cholangiocarcinoma may also be classified by growth pattern as mass-forming, intraductal infiltrative, or periductal infiltrative. Surgical resection is the best-established treatment for cholangiocarcinoma and remains the only option with curative intent [1,6,7].

Locoregional therapy is significantly less studied in cholangiocarcinoma compared to HCC; however, there is increasing evidence that its use in conjunction with systemic therapies for treatment of intrahepatic disease may prolong survival or downstage patients to allow for curative-intent resection. Hilar and distal disease are usually not approached with locoregional therapies [1,7,8].

### 1.3. Other Histologic Types

HCC and cholangiocarcinoma represent almost all malignant liver tumors in adults. Angiosarcoma is the third most common primary hepatic malignancy and is a rare sporadic mesenchymal neoplasm primarily seen in adults. Historically, angiosarcoma was associated with industrial exposure and Thorotrast administration. Epithelioid hepatic epithelioid hemangioendotheliomas (EHE) are extremely rare malignant vascular neoplasms that present as large liver masses in adults which often demonstrate indolent growth. Hepatoblastoma is the most common pediatric liver malignancy and is considered an embryonal malignancy. Undifferentiated embryonal sarcomas of the liver have also been rarely reported in children. Evidence supporting the use of locoregional therapy in these liver tumors is limited due to their rarity and is beyond the scope of this review, although the use of trans-arterial therapy has been reported in the management of pediatric hepatoblastoma [6,9]. 

## 2. Bland Embolization 

Embolization refers to therapeutic occlusion of a blood vessel through an indwelling catheter. The liver has a unique dual blood supply through the portal vein and hepatic arteries. Under normal physiology, the portal venous system supplies most of the liver’s blood supply. Both primary and metastatic liver tumors, however, derive most of their perfusion from the hepatic arterial system. This observation led to the development of arterial embolization therapies for liver tumors which aim to reduce or stop the hepatic arterial supply to the tumor [3,10].

Embolization may be performed with devices such as coils or plugs that mechanically occlude the arterial lumen, hemostatic agents that promote thrombosis, particles that occlude the smaller branches of the arterial territory, or liquid agents which promote thrombosis and/or arterial sclerosis. Mechanical devices that occlude the lumen of larger arteries or large particles are typically not effective in the management of neoplasms which are usually able to recruit collateral flow. Although historically used in the management of liver neoplasms, hemostatic agents such as GelFoam or Avitene occlude relatively large arteries and have fallen out of favor. Liquid agents such as ethanol have also been described for hepatic arterial embolization of tumors but are used less commonly. The most frequently used liquid embolic is ethanol mixed with lipiodol, a radiopaque lipophilic contrast agent that serves as an embolic as well [3,11].

Embolization of branch hepatic arteries with microparticles (particulate embolization) is also frequently performed. Further discussion of outcomes primarily refers to particulate embolization rather than the use of liquid embolic agents. Microparticles range in size from 50 to 1000 micrometers (µm) and occlude the arterioles within the neovascularized tumor. Various microparticles have been described, most of which are manufactured from polyvinyl alcohol (PVA) or polyethylene glycol (PEG). Brand names include EmboSpheres and EmboZenes [3,11].

Because embolization is also commonly performed in combination with intra-arterial chemotherapy as chemoembolization, it is common to specify bland embolization when chemotherapy is not being administered. This is also referred to as trans-arterial embolization (TAE).

### 2.1. Indications and Contraindications

Bland embolization has primarily been studied in the management of hepatocellular carcinoma. It is indicated for the treatment of BCLC intermediate-stage HCC as an alternative to chemoembolization or selective internal radiotherapy. It may also be offered for the treatment of advanced-stage HCC or unresectable intrahepatic cholangiocarcinoma, usually in conjunction with systemic therapy [1,2,7,12,13].

There are no absolute contraindications to hepatic arterial embolization other than uncorrectable coagulopathy or an inability to cannulate the hepatic artery. Ideally, platelet count should be greater than 20 k and International Normalized Ration (INR) less than 2.0 prior to arterial puncture. Embolization is considered a low-bleeding-risk intervention under the Society for Interventional Radiology (SIR) classification as there is no arterial angioplasty, stenting, or other form of intravascular device deployment [14].

Technical factors preventing selection of the hepatic artery are rarely encountered when all options for arterial access are considered. Some operators prefer non-invasive vascular imaging such as Computed Tomography (CT) Angiogram (CTA) of the abdomen and pelvis prior to arterial embolization [15].

Arterial administration of iodinated contrast agents has been associated with contrast-induced nephropathy, although the precise relationship between contrast and acute kidney injury is unclear. Most operators consider renal insufficiency to be relative contraindication for hepatic arterial embolization. Typical practice would be to pursue a risk–benefit discussion with the patient’s treatment team if there is a history of chronic kidney disease (CKD) with a baseline glomerular filtration rate (GFR) less than 30 and to defer embolization therapies in the setting of acute kidney injury (AKI) [16].

Prior allergic reaction to iodinated contrast agents may also be a contraindication to the procedure. The American College of Radiology (ACR) contrast manual is a commonly used guideline for classifying and managing contrast reactions. In general, only allergic-type reactions such as urticaria, erythema, facial or airway edema, and anaphylaxis are associated with a future risk of adverse contrast reaction. Anaphylaxis is an absolute contraindication to the procedure, whereas less severe allergic-type reactions are a relative contraindication. Consensus guidelines agree that patients with mild and moderate severity allergic-type reactions can be safely administered arterial contrast following premedication [16].

Poor liver function is a relative contraindication to hepatic arterial embolization as the procedure may trigger fulminant liver failure. Cirrhotic liver is relatively more dependent on arterial perfusion compared to normal liver. Most operators do not offer the procedure to patients with Child C cirrhosis. Patients with end-stage liver disease are often more likely to experience death from complications of decompensated cirrhosis than from tumor-related events [3,5].

Biliary obstruction is a relative contraindication to embolization as patients with obstructed biliary systems are at an increased risk of liver failure following hepatic arterial embolization. In such cases, biliary decompression with retrograde stenting or percutaneous drainage is typically performed prior to embolization. Biliary instrumentation does increase the risk of biliary sepsis with hepatic arterial embolization, although this complication remains uncommon with the use of prophylactic antibiotics [3,15].

Portal vein thrombosis (PVT) is associated with cirrhosis and is felt to cause increased dependence of the liver on hepatic arterial perfusion due to occlusion of the normal portal inflow. Most operators consider PVT a relative contraindication to hepatic arterial embolization [3,5,13].

Embolization may be offered to patients with large liver masses and possibly also to patients with infiltrative hepatocellular carcinoma. Although it is not possible to safely treat both hepatic lobes in one setting, staged embolization over two or more sessions may be offered to patients with bi-lobar disease [3,5,13].

Recurrence is not a contraindication to embolization. Microparticles do not occlude macroscopic arteries to preclude future treatment, and re-treatment of a lesion is not associated with higher risks of complications, assuming there is a lack of progression in underlying liver disease. Embolization may also be offered in patients who have previously undergone surgical resection or other locoregional therapies including radioembolization and external beam radiotherapy [5,12].

### 2.2. Procedure

Prior to embolization, a pre-procedure clinic visit is performed to assess the patient’s candidacy for the embolization and cardiopulmonary status if sedation is planned. Recent cross-sectional liver imaging is important in procedure planning and should be available at the time of the pre-procedure clinic visit. Most operators do not feel focused vascular testing such as the ankle-brachial index would be necessary as part of routine workup for arterial embolization procedures [3,10].

Arterial access is obtained, which may be either femoral, radial, or brachial. The procedure may be performed under local anesthesia only, anxiolysis, moderate sedation, or (rarely) general anesthesia. Most patients and operators prefer anxiolysis or moderate sedation [3]. Although evidence for antibiotic prophylaxis is lacking, expert consensus supports administering intravenous antibiotics at the start of the procedure. The choice of prophylactic medication depends on local preferences, but agents with gram-negative coverage are usually given [17].

A selective angiographic catheter is used to cannulate the celiac (or superior mesenteric, if variant anatomy is noted) artery and a microcatheter is used to select the hepatic arterial branch supplying the tumor. Angiography is performed to confirm selection of the appropriate artery. Cone-beam CT is valuable in confirming appropriate selections but not mandatory for embolization procedures. Microparticles are then infused through the arterial catheter until stasis of flow is obtained [3,15]. Some operators prefer to use a balloon occlusion catheter to administer embolic, which may improve the distribution and concentration of microparticles. This is often referred to as the “balloon” technique [18,19]. The arterial puncture site is closed with manual pressure or an arterial closure device.

Patients may be discharged home on the day of procedure, but many institutions prefer overnight admission for observation and expectant management of post-procedure symptoms. Follow-up imaging is recommended in 8-12 weeks to assess the treatment response. A follow-up clinic visit is also usually performed to discuss the results of embolization [3,15].

### 2.3. Complications

Particulate embolization causes ischemia of the treated tumor which triggers an inflammatory response that clinically manifests as post-embolization syndrome. Post-embolization syndrome is seen in almost all patients and is characterized by abdominal pain (often right upper quadrant), nausea, vomiting, fevers, and chills. Symptoms resolve over 3–7 days and without permanent sequelae. It is managed symptomatically with antiemetics and analgesics, although admission for pain management and intravenous hydration is not uncommon [3,6,11].

Clinically significant complications of hepatic arterial embolization are uncommon. Reported complication rates vary, but it is estimated that ~5% of patients will experience a complication requiring treatment or unexpected admission. Data on precise rates of specific complications are scant [11].

The liver depends increasingly on hepatic arterial blood supply as cirrhosis progresses. Decreased liver function may be seen following embolization. While transient elevations in liver function tests (LFTs) or increased bilirubin are common, fulminant liver failure is also possible, especially in patients who already have end-stage liver failure or PVT [3,11].

Non-target embolization, also referred to as off-target embolization, refers to unintended treatment of nearby structures causing injury or clinical symptoms. Embolization of the gallbladder, stomach, pancreas, small bowel, and right diaphragm are possible during the treatment of the hepatic arteries. While some degree of non-target embolization is not uncommon, symptomatic—let alone clinically significant—non-target embolization is very rare with proper angiographic techniques. Acute ischemic cholecystitis due to off-target embolization may require emergent surgery. Gastric or duodenal/jejunal embolization may rarely lead to ischemic ulcers complicated by bleeding or even perforation [3,15].

Infectious complications of hepatic arterial embolization are also rare. Infection of necrotic tumor may lead to the development of an intrahepatic abscess requiring antibiotic treatment or percutaneous drainage. The imaging appearance of intrahepatic abscesses overlaps that of expected ischemia during the first ~2 weeks after embolization, and liver abscess is primarily a clinical diagnosis during this period. Signs of abscess include persistent or high-grade fever and general clinical decompensation [3].

Any arterial puncture carries the risk of hematoma, pseudoaneurysm or dissection. Pseudoaneurysms complicate approximately 2–5% of femoral arterial punctures and in most cases are readily treatable with percutaneous thrombin injection. Exsanguinating hemorrhage or acute limb ischemia requiring emergent vascular surgery is exceedingly rare. While transient vasospasm is relatively common, true endovascular arterial injury such as dissection, perforation, or untreatable thrombosis is rare during embolization. Complications related to contrast administration, including contrast reactions and acute kidney injury may also occur [3,10].

### 2.4. Outcomes

Assessing the outcome of any oncologic treatment can be performed using clinical and radiologic response criteria. The most common clinical criterion is mortality, which is often assessed though overall survival (OS). Standardized imaging-based assessments of treatment response have been developed. The Modified Response Criteria in Solid Tumors (mRECIST) is an internationally recognized system for assessing treatment response on imaging which has been validated in HCC as well as cholangiocarcinoma. mRECIST defines standard criteria for complete response, partial response, stable disease, and progressive disease based on measurements of up to two intrahepatic lesions in addition to measurements of any lymph nodal or remote metastases. Given the high mortality rates of patients with HCC, most studies of locoregional therapy rely on mRECIST to assess treatment response, either directly using rates of complete response as their primary endpoint or indirectly by using progression-free survival (PFS) as an endpoint. PFS is defined as the interval between treatment and clinical progression based on objective criteria such as mRECIST [20].

The level of evidence for benefit for bland embolization is relatively low, consisting primarily of retrospective data, but there is considerable clinical experience with its use in primary hepatobiliary tumors. Published results support the effectiveness of embolization at delaying radiologic signs of progression and—to a lesser extent—improving survival in patients with HCC. Progression-free survival following bland embolization is approximately 4-6 months. Unfortunately, recurrence is common either in the treated region or elsewhere in the liver [3,15].

## 3. Chemoembolization

Chemoembolization combines hepatic arterial embolization with intra-arterial chemotherapy administration. It may also be referred to as trans-arterial embolization (TACE). The addition of chemotherapy is hypothesized to potentiate embolization through cytotoxic effects. Conventional cytotoxic chemotherapeutic agents are used, most commonly doxorubicin (Adriamycin), although platinum-based agents have also been reported. Chemoembolization was initially described with liquid embolic emulsion which is referred to as “conventional” chemoembolization (c-TACE). The procedure may also be performed with drug-eluting microparticles as drug-eluting bead chemoembolization (DEB-TACE) [3,6,15].

### 3.1. Indications and Contraindications

TACE, like bland embolization, has been most extensively investigated in the treatment of HCC. Its primary indication is in the management of BCLC intermediate-stage disease, and is often performed as initial therapy for such patients. It may also be offered to patients with advanced-stage HCC, usually in combination with systemic therapy. TACE is offered as a part of palliative or neoadjuvant therapy for unresectable intrahepatic cholangiocarcinoma [1,4,5,7,13].

The contraindications from chemoembolization are the same as those for bland embolization, as most are generic to the arterial embolization process. Like bland embolization, chemoembolization may be offered to patients who have previously undergone locoregional therapies, including SBRT, chemoembolization, bland embolization, or radioembolization [3,12].

### 3.2. Procedure

Pre-procedure evaluation is identical for TACE and bland embolization. Arterial access and selection are also substantially similar. For c-TACE, a mixture of lipiodol and chemotherapeutic agent is infused into the hepatic arterial branch of interest until stasis of flow is observed. Lipiodol, or ethiodized oil, is a viscous lipophilic contrast agent with embolic properties. Its radiodensity allows for real-time fluoroscopic confirmation that that appropriate area has been embolized and that no significant non-target embolization has occurred. Some operators prefer to administer chemoembolic emulsion through a balloon occlusion catheter, which is also known as “balloon TACE” (b-TACE) [19]. Because it permanently and densely opacifies the treated area, lipiodol administration limits the CT assessment of treatment response [3].

The procedure for DEB-TACE is identical to that for bland microparticle embolization. Drug-eluting beads are essentially microparticles impregnated with chemotherapeutic agent, usually doxorubicin. The b-TACE technique can also be applied to DEB-TACE. Various forms of drug-eluting particles are manufactured commercially [3].

Patients are followed up clinically and with repeat imaging, typically 8–12 weeks after treatment. TACE is then repeated if there is evidence of residual viable tumor or new HCC lesion. Additional treatments are offered as a residual or new tumor is detected. This conventional practice of offering multiple treatments is sometimes referred to as “on-demand” TACE to emphasize that patients may require multiple treatments [3,15].

### 3.3. Complications

Chemoembolization complications are the same as those of bland embolization. There is no consistent evidence that the addition of chemotherapy significantly increases the risk of major complications including liver failure or hepatic abscess [21,22]. The preference for chemoembolization or bland embolization is often operator-dependent; most institutions prefer chemoembolization over bland embolization for the treatment of HCC or cholangiocarcinoma. An argument may be made that the addition of chemotherapy adds potential benefits without proven additional risk.

### 3.4. Outcomes

Evidence supporting the use of TACE in HCC is primarily derived from retrospective studies with few randomized trials. The overall weight of evidence supports its benefit in increasing survival in patients with intermediate-stage HCC [5,10,13]. Although different values have been provided, an estimate would be increasing median survival from 2 months with supportive care to 8 months with TACE [15].

Randomized studies do support the effectiveness of TACE in producing objective radiologic responses and improved PFS compared to supportive therapy [23,24]. When retrospective studies and radiologic-based outcomes are not weighed, a dissenting view may be considered that TACE and bland embolization have not been shown to increase survival in patients with HCC [25]. The baseline high mortality of patients with intermediate-stage HCC limits the ability of clinical trials to demonstrate mortality benefit in the absence of a dramatic treatment effect.

There is considerably less evidence on the effectiveness of TACE in the management of cholangiocarcinoma. Retrospective studies suggest a survival benefit in patients with unresectable intrahepatic cholangiocarcinoma with reported overall survival of 9–12 months after TACE [8].

Comparing TACE with other locoregional or systemic therapies is difficult due to a lack of clinical trials and a relative lack of standardization in TACE techniques. Several randomized trials reported delayed time to progression and a higher rate of complete treatment response with TACE compared to bland embolization, although they were not designed to compare mortality [21,22].

Several clinical trials, including a few randomized trials, have compared c-TACE and DEB-TACE. These have not consistently reported the improved mortality of treatment response with DEB-TACE, although a recently published prospective randomized trial did report improved overall survival with DEB-TACE [26]. Clinical trials have consistently reported reduced severity of post-embolization syndrome with DEB-TACE, as well as, however, a reduced need for retreatment [27,28,29,30].

The use of balloon occlusion TACE techniques may improve embolic dose distribution compared to the use of conventional microcatheters in both c-TACE or DEB-TACE, although this has not been validated in rigorous trials [18,19,31].

## 4. Radioembolization

Radioembolization (TARE) or selective internal radiotherapy (SIRT) refers to intra-arterial administration of radioactive materials for brachytherapy. Selective angiography allows for a less invasive administration of brachytherapy compared to surgery or image-guided percutaneous methods; however, the employment of radioembolization was historically limited by the difficulty of developing an appropriate delivery method for the radioactive agent [6,10].

Yttrium-90 (Y-90) microspheres are the only type of radioembolization agent currently used in clinical practice. These are resin (SIR-Sphere) or glass (TheraSphere) microparticles approximately 20–60 µm in size, impregnated with the radiotherapeutic Y-90. Y-90 microparticles are slightly smaller than the 100 µm particles used in bland embolization or DEB-TACE. Y-90 is a beta emitter with a half-life of 64.1 h. Beta particles are equivalent to electrons penetrating only a few centimeters of soft tissue, allowing for targeted delivery of ionizing radiation to the treated area of liver with a reduced dose to the surrounding tissues. The microparticles embolize in the capillaries of the liver, permanently depositing within the parenchyma and administering the dose to the vascular territory of the selected artery [6,15].

Although microparticles cause embolic effects, the therapeutic effect of Y-90 microsphere arterial embolization is primarily mediated through ionizing radiation as a form of brachytherapy rather than ischemia. As a result, some authors prefer the SIRT terminology over radioembolization/TARE [6].

**Figure 1:**
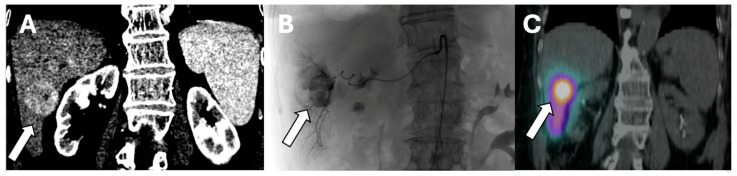
64-year-old male patient with a right hepatic HCC who underwent Y-90 radiation segmentectomy treatment. (**A**) Pre-procedure CT demonstrating arterially enhancing right hepatic lobe HCC (arrow), (**B**) intraprocedural arteriogram demonstrating the lesion (arrow) on fluoroscopy during the Y-90 treatment session, and (**C**) post-treatment SPECT CT demonstrating Y-90 activity deposited in the tumor (arrow).

SIRT can be applied selectively to one or two segments of the liver or to an entire lobe. Segmental administration or radio-segmentectomy is performed with a higher dose to ablate the treated area, while lobar administration is performed with a lower dose intending to arrest tumor growth and induce hypertrophy of the remaining liver segments. This distinction is important when planning SIRT treatments [32]. Figure 1 is an example of Y-90 radiation segmentectomy treatment.

### 4.1. Indications and Contraindications

Like other locoregional therapies, SIRT has primarily been evaluated for the treatment of HCC. The therapeutic intent of SIRT depends on the size of the treated HCC. Radio-segmentectomy is indicated for treatment of BCLC early-stage or intermediate-stage HCC involving one or two segments. Lesions involving more than two segments cannot be ablated due to the risk of liver failure [15,32].

SIRT for larger HCC lesions involves lobar treatments. Lobar SIRT is indicated for intermediate-stage HCC and may also be selectively offered to patients with advanced-stage disease. Tumors involving a single lobe are approached with uni-lobar SIRT to the affected side. More extensive tumors can be managed with staged treatment of the more affected lobe followed by treatment to the contralateral lobe in 4–8 weeks. SIRT may also be offered to patients with diffuse infiltrating HCC [6,11].

SIRT is also indicated for the palliative or neoadjuvant treatment of unresectable intrahepatic cholangiocarcinoma playing a similar role to TACE [8].

General contraindications to angiography are the same for bland embolization, TACE, and SIRT. The dose of embolic particles is significantly smaller in SIRT compared to other methods with much less tissue ischemia. As a result, post embolization syndrome is considerably milder. Whereas overnight admission for analgesia is frequently needed for embolization or chemoembolization, nausea/vomiting and pain after SIRT are easily managed on an outpatient basis with oral medication [6,33].

Another consequence of reduced ischemia is lower rates of liver function decompensation compared to embolization or chemoembolization. As a result, SIRT is usually the preferred trans-arterial therapy in patients with poor liver function or PVT who are at increased risk of fulminant liver failure following arterial embolization. The risk of liver abscess formation is also felt to be lower with SIRT compared to other trans-arterial therapies due to decreased tissue ischemia. The risk of liver failure is felt to be increased in patients with underlying biliary obstruction; in such cases, decompression of the biliary system is pursued prior to SIRT [6,15].

The effects of non-target treatment somewhat vary with SIRT compared to embolization and chemoembolization. Experience suggests a minimal risk of cholecystitis with radioembolization of the cystic artery. Radioembolization of the stomach or small bowel can cause severe ulceration or chronic gastritis/duodenitis. Radioembolization of the pancreatic head has rarely been associated with pancreatitis and radioembolization of the falciform artery has rarely been associated with skin ulceration. The lungs are highly sensitive to radioembolization, and radiation pneumonitis may develop if there is significant shunting of flow from the hepatic arteries to the lung. Because lung shunting or non-target perfusion to the gastrointestinal tract are major contraindications, patients must obtain a hepatic arterial perfusion study prior to SIRT [5,6].

Unlike bland embolization or chemoembolization, prior radiotherapy is a contraindication to SIRT. As a result, patients cannot be “re-treated” for recurrence. Additionally, a history of prior radiotherapy to the abdomen or nearby organs may also preclude SIRT, especially if non-target effects are seen [6,15].

### 4.2. Procedure

Prior to treatment, patients are evaluated in the clinic to assess candidacy. Imaging must be carefully reviewed to select the appropriate area to treat. A multidisciplinary consultation involving radiologists, radiation oncologists, and medical oncologists treating the patient is strongly recommended [1,5,13]. If the mass involves one or two hepatic segments, the patient is referred for radiosegmentectomy, whereas uni-lobar or bi-lobar treatment is planned for larger lesions. Some operators prefer to obtain a CT angiogram of the abdomen for pre-procedure planning, although this is not mandatory [15,34].

Once a treatment target is chosen, the patient is scheduled for angiography with hepatic arterial perfusion study to exclude lung shunt or non-target perfusion. Arterial access is obtained, and hepatic arteriography is performed to select the appropriate hepatic arterial branch. If radiosegmentectomy is being pursued, superselective angiography is attempted to identify an appropriate arterial branch supplying the lesion. Uncommonly, variant arterial anatomy will preclude radiosegmentectomy in which case, lobar treatment is chosen. If lobar treatment is pursued, the right or left hepatic artery is selected. Coil embolization of arterial branches potentially causing off target treatment is performed if high-risk anatomic variation is seen. Traditionally, routine embolization of the right gastric, gastroduodenal, and falciform arteries was performed to prevent non-target treatment, but more recent evidence suggests that selectively embolizing collaterals in high-risk anatomic situations is also safe. Technetium-99 m labeled macro-aggregated albumin (MAA) is then infused through a microcatheter in the appropriate hepatic artery. The procedure is completed, and the arterial puncture site is closed [34].

Immediately after the MAA injection procedure, the patient is transferred to nuclear medicine imaging for examination. Most institutions perform imaging with a gamma camera for planar images and SPECT/CT, although only planar images are mandatory to exclude lung shunt. MAA particles measure ~10–90 µm, which is similar in size to 20–60 µm Y-90 microspheres, and embolize in the hepatic tissues, allowing for a reasonable simulation of where a Y-90 dose would be distributed [11,34].

Gamma cameras detect the gamma particle emitted by the Tc-99 m; the dose of Tc-99 m administered is much less than the therapeutic dose of Y-90 administered for SIRT and does not cause tissue effects. This allows for the localization of the administered dose and determination of non-target perfusion. The lung shunt fraction is calculated by dividing the counts in the lung fields by the total lung and liver counts on planar imaging. Additionally, perfusion to the stomach, pancreas, bowel, or skin is identified by visual inspection. High lung shunt fraction or observed non-target perfusion is a contraindication to SIRT. If this is seen, the patient is typically treated with bland embolization or chemoembolization instead [34].

Once the MAA perfusion study is performed, an appropriate dose of Y-90 particles is calculated. Details of SIRT dosimetry is beyond the scope of this review, but there are three general approaches: standard, multi-compartment, and image-based. Standard dosimetry employs body surface area and liver mass to calculate the dose, whereas multicompartment models use data from MAA planning studies on target dose, non-target liver dose, and lung dose. Image-based methods, also known as personalized dosimetry, apply computational methods to 3D information obtained during planning SPECT-CT to calculate an optimal dose. Imaging-based methods require significantly more computational power but allow for the administration of higher doses. The lung shunt fraction is critical to dose calculation because lung dose cannot exceed 30 Gray (Gy) in one setting or 50 Gy over multiple treatments [35].

After the appropriate dose is selected, the patient returns for the actual radioembolization procedure. This is performed identically to the MAA perfusion study, except that Y-90 microspheres are administered to the appropriate hepatic artery instead. Many institutions will perform immediate nuclear imaging of the patient with either SPECT-CT or PET to confirm appropriate dose distribution, although this is not strictly mandatory. The patient is usually discharged the same day. If bi-lobar treatment is pursued, the process of MAA perfusion study is repeated on the contralateral side followed by completion of treatment with contralateral SIRT. Once treatment is completed, follow-up imaging is performed with CT or MRI of the liver to assess the treatment response [6,15].

### 4.3. Outcomes

SIRT has primarily been evaluated as a treatment for HCC. There is a relative lack of prospective trials demonstrating the effectiveness of SIRT; however, these do support its effectiveness in producing objective treatment responses and delaying the progression of disease in HCC. Retrospective trials suggest 60–80% objective response rates (ORR) based on follow up imaging and an overall increase in survival for patients with intermediate-stage HCC [36,37]. These outcomes have also been noted retrospectively in the author’s institutional experience [38]. SIRT has also been associated with increased survival in patients with intrahepatic cholangiocarcinoma, with overall survival reported as 8–22 months among retrospective studies [8,39].

Among SIRT techniques, radiosegmentectomy is associated with a decreased risk of recurrence. Retrospective data suggest that radiosegmentectomy may offer similar outcomes to surgery or ablative techniques with a median survival of 6.7 years and may be considered an alternative to resection or ablation [32].

The use of image-based (personalized) dosimetry may be associated with improved outcomes compared to standard dosimetry. A single prospective trial reported improved response to treatment with image-based compared to standardized dosimetry [40].

There are little data available to compare SIRT with other locoregional therapies. The overall survival rates of TACE and SIRT are similar, although SIRT can improve the quality of life, especially if fewer treatment sessions are necessary [33]. One randomized trial reported significantly increased time to progression in patients with HCC undergoing SIRT compared to TACE, although the population size was small [36].

## 5. Ablative Therapies

Ablation is a class of minimally invasive therapies that use directed application of energy or medication to treat tumors. Most ablative therapies are performed percutaneously using specialized probes under real-time imaging guidance. The most-studied ablative therapies are radiofrequency ablation (RFA), microwave ablation (MWA), ethanol ablation (percutaneous ethanol injection, PEI), and cryoablation. These have been shown to produce similar outcomes compared to surgical resection for patients with small HCCs less than 3 cm in size. As a result, ablation is indicated as a primary treatment for BCLC early stage HCC [1,4,5,13,41].

### 5.1. Radiofrequency Ablation

RFA uses electromagnetic energy to heat a targeted area of tissue. Thermal injury and coagulation destroy the targeted tumor. RFA is administered using one or more electrodes inserted into the lesion of interest. Electrodes come in many different designs, but the common factor is that they can be inserted percutaneously either directly using a sharp needle tip or through a trocar. A technical description of RFA probe design is beyond the scope of this review, but most probes are intended to thermally ablate a region approximately 5 cm in diameter, effectively treating tumors up to 3 cm in size [42].

RFA is indicated to treat BCLC early stage HCC and may be considered alongside other locoregional therapies in the management of intermediate-stage disease. It is also indicated for treatment of unresectable intrahepatic cholangiocarcinoma [7,42].

The only absolute contraindication to RFA is uncorrectable coagulopathy. Most operators prefer a platelet count greater than 50 k and an INR less than 1.5 due to the relatively increased bleeding risk in the setting of visceral organ puncture [14]. Lesion location in the periphery of the liver is a strong relative contraindication if the anticipated ablation zone would involve the diaphragm, stomach, or bowel. Similarly, a location near the hepatic hilum is a strong relative contraindication due to the risk of biliary or vascular injury. A subcapsular location is a weaker relative contraindication due to the risk of bleeding or pain [42].

Nearby blood vessels can reduce the effectiveness of RFA through the *heat sink effect*, where heat is transferred to the blood leading to less energy deposition into the targeted lesion and suboptimal ablation. As a result, lesions close to portal or hepatic veins are relatively contraindicated, although RFA may still be attempted [11,42].

Although ablation with multiple probes can be attempted for even large masses, the effectiveness of RFA considerably decreases with lesion size. Most authorities consider a tumor greater than 5cm in size a strong relative contraindication to RFA [42].

Typically, RFA is performed under general anesthesia or deep sedation as the procedure causes significant pain. Probe insertion can be performed with either CT or ultrasound guidance based on operator preference and which modality better demonstrates the lesion. Some operators perform a biopsy immediately prior to ablation. Once the probes are in satisfactory procedure, ablation is performed. Ablation of the tract used to approach the tumor has been proposed to reduce seeding, although there is limited evidence to support this practice. The procedure is typically performed on an outpatient basis, with follow up imaging performed in 8–12 weeks to assess treatment response [42].

Complications of RFA are uncommon and typically relate to anatomic considerations. Major complication rates are estimated at ~5%. Pneumothorax may occur if the pleura is traversed during the procedure. Hemothorax may be seen with intercostal artery injury. Bowel perforation has been reported either from thermal damage during ablation of peripheral lesions or from traversing bowel with a probe. Gallbladder perforation and biliary stricture may also occur. Hemoperitoneum may occur from post-ablation hemorrhage. In most cases, careful pre-procedure evaluation and attention to intra-procedure US or CT can prevent such complications [15,42].

Tumor seeding has long been a concern with ablative therapies and is used as a justification for offering first-line resection. Clinical experience suggests this is not as significant a risk as previously believed. Tumor seeding has been only rarely reported, with less than 0.5% of ablations affected [42].

### 5.2. Microwave Ablation

MWA uses high-frequency microwaves to cause thermal damage to a tumor in a similar manner to RFA. MWA is also administered using probes; however, a grounding pad is not needed. Numerous designs for MWA probes exist and lesions can be targeted with one or more probes for ablation. Compared to RFA, MWA probes offer better ablation of tissues that are poor heat conductors and greater control of the ablation zone. As a result, MWA is often thought of as an improvement over RFA and is gradually replacing it in clinical practice [5,42].

The indications for RFA and MWA are identical, with strong clinical evidence supporting their equivalency in the treatment of HCC or other small liver tumors. Like RFA, MWA is of decreasing effectiveness with increased tumor size and usually not attempted on lesions greater than 5 cm in size [42].

The procedural approach to MWA is substantially like that for RFA. Both require general anesthesia or deep sedation and are usually performed on an outpatient basis. The MWA probes are inserted into the lesion and ablation is initiated. Follow-up imaging is performed to assess the treatment response. Complications of MWA are also identical to those of RFA [42].

### 5.3. Cryoablation

Cryoablation employs rapid cooling to damage tumor cells. Although various cryoablation probe designs exist, all rely on the Joule–Thomson effect to rapidly cool tissues. Several freeze–thaw cycles must be applied for adequate treatment of a lesion. Various combinations of cryoablation probes can be applied to treat small or large tumors [43].

Like other ablative therapies, the only absolute contraindication is irreversible coagulopathy; however, cryoablation is relatively contraindicated in peripheral and hilar lesions due to the risk of tissue damage to surrounding organs [14,43].

Compared to RFA, increased lesion size is less of a contraindication to cryoablation, as probe combinations can be used to treat lesions up to 9cm size. Cryoablation is also less subject to *heat sink* effects, which makes it relatively more effective for lesions near blood vessels or fluid filled structures [15,43].

The procedural outline for cryoablation, MWA and RFA is similar: ablative probes are inserted into the lesion of interest under CT or ultrasound guidance. Cryoablation is considerably less painful than RFA or MWA and can be readily performed under moderate sedation. This is a major advantage in patients who cannot tolerate general anesthesia or deep sedation. Another technical advantage of cryoablation is the ability to visualize the ablation zone on CT as a hypodense “ice ball”, providing real-time confirmation of technical success. Cryoablation is usually performed as an outpatient procedure [43].

All ablative therapies carry general risks of tissue damage to nearby anatomic structures due to thermal injury or direct perforation with the probe. Compared to RFA or MWA, cryoablation has been reported to carry a higher complication rate when treating liver tumors. This is primarily driven by the increased risk of hemorrhage; RFA and MWA have coagulative effects, whereas cryoablation does not independently promote hemostasis [43].

A rare but serious risk of hepatic cryoablation is “cryoshock”, which is defined as hypotension, multiorgan failure, and thrombocytopenia. The exact prevalence of cryoshock is unknown but it is believed to be less than 1%. Cryoshock is associated with significant mortality from organ failure and is difficult to manage. Ablation of large tumors may carry a higher risk of cryoshock. Although similar reactions have been extremely rarely reported following other thermal ablation techniques, the possibility of cryoshock has led some institutions to abandon cryoablation of liver tumors in favor of MWA or RFA [43].

### 5.4. Ethanol Ablation

Chemical ablative therapies use directed administration of caustic or reactive substances to cause localized tissue damage. Ethanol and acetic acid are the only chemical ablation agents with significant experience in the treatment of liver tumors.

Ultrasound-guided ethanol ablation (percutaneous ethanol ablation, PEI) for hepatocellular carcinoma was initially developed in the 1980s and represented the first oncologic ablative therapy. Seminal clinical trials in the early 1990s showed similar recurrence and mortality rates compared to surgical resection, opening the era of percutaneous tumor ablation. Ablation is performed by directly injecting ethanol with a needle into the lesion of interest under ultrasound (or much less commonly, CT) guidance. Although the procedure is well tolerated under local anesthesia or anxiolysis, only a small volume may be injected in a single session due to systemic toxicity. Acetic acid ablation is essentially identical to ethanol ablation in technique, with the substitution of concentrated acetic acid for ethanol, and provides similar outcomes. Complete treatment of an early stage HCC measuring 1–3 cm requires ~4–8 staged sessions which are difficult to coordinate. As a result, chemical ablation has been almost completely supplanted by RFA, MWA and cryoablation techniques, which can achieve the same effect in a single procedure with similar risk profiles [41].

### 5.5. Outcomes of Ablative Therapies

There is robust clinical evidence, including prospective and randomized trials, to support the effectiveness of MWA, RFA, Cryoablation, and PEI in the management of BCLC early-stage HCC. Prospective overall survival rates are similar between ablation and surgical resection, with ~30% 3-year mortality. Ablative therapies carry a lower risk of major procedural complications compared to surgery [2,5,13]. Retrospective data including the author’s institutional experience also support their effectiveness [44]. Major clinical guidelines endorse surgical resection or ablation as the preferred initial treatment of early-stage HCC [2,5,13]. The effectiveness of ablation for intermediate-stage HCC is less clear, but it has been reported as a potentially effective salvage therapy after TACE or SIRT [12].

Increasing experience supports the use of ablative therapies in mass-forming intrahepatic cholangiocarcinoma, and they are now included in newer treatment guidelines if patients are not candidates for surgical resection [7], although overall survival rates widely vary from 9 to 32 months [8].

## 6. Future Directions

### 6.1. Improved Dosimetry for SIRT

In addition to the development of personalized dosimetry, novel radiopharmaceuticals are being developed which may better simulate Y-90 microspheres. The recent literature has raised questions on the extent to which injection of Tc-99 m MAA adequately approximates the injection of Y-90 microspheres. Although similar in size, albumin particles in MAA are chemically different from microspheres and show greater variability in size. Microparticles containing Holmium-166 (Ho-166) and Rhenium-188 (Re-188) have been developed which may be used for planning and dosimetry instead of MAA. Although primarily beta emitters, both Ho-166 and Re-188 produce significant gamma radiation, allowing for accurate dosimetry using conventional gamma cameras. This may allow for further improvements in dosimetry, allowing for the administration of potentially more effective radiotherapeutic doses [35].

### 6.2. Selective Internal Radiotherapy with New Radiotherapeutics

Alpha particles have an even shorter attenuation length and cause greater ionization effects, potentially increasing the dose delivered to the embolized tissue while reducing the effects on nearby organs. As a result of these theoretical benefits, there is considerable active research towards developing microparticles impregnated with an alpha emitter [45].

Additionally, there is interest in developing microparticles with beta emitters other than Y-90. Ho-166 and Re-188 microspheres have been proposed for use as the primary therapeutic agent in addition to serving as an alternative to Tc-99 m MAA for dosimetry [35].

### 6.3. Controlling Injection Parameters during SIRT

It is known from computational models that distribution of microparticles varies significantly based on the injection parameters such as duration of injection, volume injected, and pressure applied [46]. Additionally, microcatheter types used can vary significantly in injection properties. Current practice does not standardize injection parameters during SIRT, and more research is needed to define optimum injection technique [47].

### 6.4. Irreversible Electroporation

Electroporation refers to the application of electromagnetic pulses to form holes in cell membranes. Irreversible electroporation (IRE) uses electromagnetic fields applied through percutaneously placed electrodes to cause permanent loss of cell membrane integrity in a treatment zone. Unlike thermal or chemical methods of ablation, IRE causes minimal structural damage. Because IRE is unlikely to disrupt nearby vascular structures, it is a promising treatment modality for hilar liver tumors which are otherwise not amenable to ablation or resection due to surrounding biliary, arterial, and portal venous structures [48].

Conceptually, IRE electrodes are like other ablation probes and placed into the tumor or interest under CT or ultrasound guidance. Neuromuscular blockade is necessary because the electromagnetic pulses applied can cause severe muscle contractions; therefore, the procedure can only be performed under general anesthesia. The risks of IRE overlap significantly with other ablative therapies and relate to inadvertent perforation of nearby organs by the ablation probe(s) [48].

Most of the IRE literature deals with unresectable pancreatic adenocarcinoma; however, it has also been reported as a potential treatment for primary or metastatic liver tumors. Outcomes remain to be seen, and IRE should be considered as a semi-experimental treatment modality [48].

### 6.5. Focused Ultrasound (HIFU)

High-intensity focused ultrasound (HIFU) applies ultrasound pulses to cause localized tissue heating or cellular damage. Unlike other ablative therapies, HIFU is applied externally (extracorporeal administration) and is completely non-invasive. Although HIFU technologies have existed for decades, problems with effectively applying mechanic energy with ultrasound (“coupling”) and reliably focusing its deposition to a targeted organ have prevented its clinical application until recently. These technical issues in HIFU research have led to the development of numerous variations in HIFU including contrast-enhanced HIFU and histotripsy [49].

HIFU remains an emerging technology. Although there is increasing experience with its use in humans for ablation of liver lesions, evidence is insufficient to support its routine use [50].

### 6.6. Combination Locoregional and Systemic Therapy

There is growing interest in combining locoregional and systemic therapies. A major clinical trial failed to show improvement by combining TACE with sorafenib in intermediate-stage HCC [51], and major guidelines do not endorse combining systemic therapy and TACE in intermediate-stage HCC [5,13]. Additional research is needed, however, to evaluate combination therapy with other agents as well as combination therapy in advanced disease. There is little published on combination therapy in cholangiocarcinoma management.

Immunotherapy has been of great importance to medical oncology and is increasingly employed in the management of solid tumors. Following the validation of immunotherapy as a systemic treatment option for HCC, there are multiple ongoing clinical trials comparing the addition of immunotherapy to TACE with TACE alone. Several of these have preliminarily reported positive results with the combination of TACE plus cadonilimab and levatinib and TACE plus durvalimumab and bevacizumab [52,53].

Thermal and chemical ablation is immunogenic and may potentiate therapeutic immune response. This has led to the proposal of combined systemic immunotherapy and local ablation to manage unresectable hepatobiliary tumors, although current research remains preclinical [54].

A related concept is intra-tumoral immunotherapy, which is the direct injection of immunotherapeutic agents into a tumor using similar techniques to percutaneous biopsy. This technique is proposed as an adjunct to systemic immunotherapy [54].

## 7. Selecting the Optimal Locoregional Therapy

The plethora of locoregional therapies may potentially create uncertainty for practicing clinical oncologists. A multidisciplinary discussion involving medical oncologists, radiation oncologists, interventional radiologists, diagnostic radiologists, and anatomic pathologists in a “tumor board” setting is often necessary (Table 1 and Table 2).

### 7.1. Hepatocellular Carcinoma

**Figure 2:**
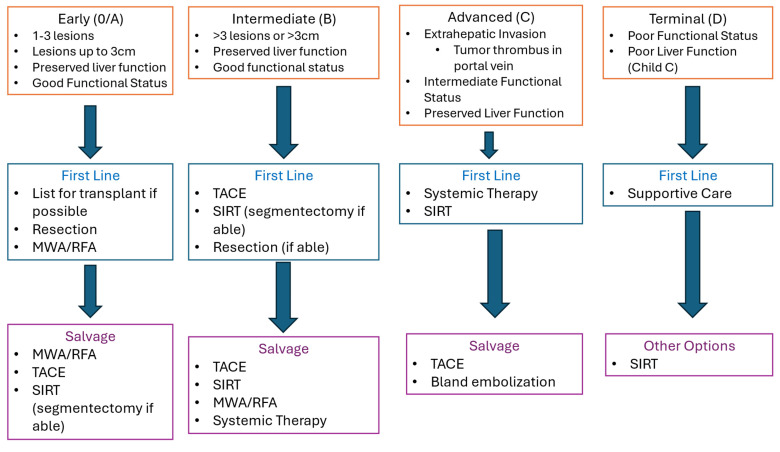
Management of HCC based on BCLC stage.

For BCLC early-stage HCC, there is a broad consensus supporting ablation or resection as the primary option in clinical management (Figure 2). Factors favoring resection are solitary lesion, peripheral or subcapsular lesion, and a lack of medical comorbidity. The presence of two or three lesions and medical comorbidities increasing surgical risk favors ablation [2,4,5]. Patients who are not candidates for resection or ablation should be evaluated for additional locoregional options with SIRT or TACE. Recurrent early-stage disease is primarily managed with percutaneous ablation.

Management of intermediate-stage HCC is more controversial, and the available evidence offers less clarity on when to offer locoregional therapy. The 2022 BCLC system recommends initial treatment with TACE for intermediate-stage disease with well-defined lesions in the absence of PVT or bi-lobar disease (Figure 2) [2]. The American Association for the Study of Liver Disease (AASLD) has recently updated its commonly referenced clinical guideline for the management of HCC in 2023. AASLD bases management of HCC on the BCLC staging system, which recommends TACE or SIRT as a primary management for intermediate-stage diseases [5]. The older European Association for the Study of the Liver (EASL) guidelines last updated in 2018 also recommends using the BCLC staging system and performing TACE for intermediate-stage disease. EASL considers SIRT as an alternative to TACE [4]. Other approaches favor SIRT as the initial treatment for intermediate-stage disease, with radio-segmentectomy preferred for unifocal lesions involving 1–2 segments and single-lobe or staged bi-lobar SIRT for multifocal or infiltrative disease. Given emerging data supporting low recurrence, radio-segmentectomy should be strongly considered whenever technically feasible as an initial management HCC with well-defined lesions > 3 cm in size involving one or two lobes.

Other treatment options for intermediate-stage HCC include systemic therapy and resection. Resection of multiple hepatic segments should also be considered, although such patients are rarely surgical candidates. Systemic therapy may be considered as an option if patients are not candidates for resection or locoregional therapy. Combining locoregional therapy with systemic therapy as initial treatment of intermediate-stage HCC is controversial; AASLD also recommends against combining locoregional and systemic treatments outside of a clinical trial [5], but new developments in immunotherapy may lead to changes in recommendations.

Management of recurrent intermediate-stage HCC is also less clear; however, TACE and ablative therapies may be used to treat foci of residual or recurrent disease. SIRT and external radiotherapies are also valuable salvage treatment options [12].

Locoregional therapy is usually considered a second-line option in patients with advanced-stage HCC who do not respond to systemic therapy. SIRT is often preferred in these cases as it may be better tolerated by patients with poor performance status and reduced liver function. Terminal disease is generally felt to be contraindication to locoregional therapy in HCC.

### 7.2. Cholangiocarcinoma

The goals of locoregional therapy for intrahepatic cholangiocarcinoma are to increase survival, palliate symptoms, and potentially decrease tumor size to allow for potentially curative resection. Locoregional therapies are best employed in conjunction with systemic therapy and may be considered in patients who cannot tolerate or do not respond to systemic therapy. Although there is limited consensus on optimal use, it is reasonable to initially offer ablation of unresectable intrahepatic tumors whenever technically possible and to consider TACE or SIRT otherwise. Unfortunately, there are limited treatment options available for hilar or extrahepatic disease [7,13,39].

## 8. Conclusions

Ablative therapies are a well-established treatment for early-stage HCC and can be offered to a wider selection of patients than surgical resection. The choice of ablative therapy depends on local preference. Trans-arterial therapies are well established in the treatment of intermediate-stage HCC, with the literature supporting both the use of TACE and SIRT. Unifocal intermediate-stage HCC may be approached with ablative therapy or trans-arterial therapy. Trans-arterial therapies also play an important role in the management of recurrent HCC, although their role in advanced-stage HCC is controversial.

Although there is less evidence supporting the use of locoregional therapies in the treatment of other primary hepatobiliary tumors, clinical experience supports the use of ablative therapies in patients with unresectable intrahepatic cholangiocarcinoma when technically feasible. Trans-arterial therapies may be combined with systemic therapy in the treatment of unresectable intrahepatic cholangiocarcinoma, either as a palliative measure or with the intent to downstage the tumor to allow surgical resection.

Advances in locoregional therapy in the past 4–5 years have focused on perfecting trans-arterial therapy, with growing evidence supporting the use of personalized dosimetry in SIRT and establishing good outcomes with radiosegmentectomy. The current research focuses on identifying the optimal methods of trans-arterial therapy. New radiopharmaceuticals and ablative technologies such as HIFU and IRE may potentially change the landscape of locoregional therapy of primary liver neoplasms in the next 5–10 years.

## Figures and Tables

**Table 1:** Overview of locoregional therapies in hepatobiliary tumors.

Locoregional Therapy Indications and Contraindications			
Technique	Description	Indications	Major Contraindications
Trans-Arterial Therapies			Irreversible coagulopathy Anaphylactic contrast reaction
Bland Embolization	Selective infusion of microparticles into hepatic artery	Intermediate-stage HCC Unresectable intrahepatic cholangiocarcinoma Recurrent HCC	Child C Cirrhosis Terminal HCC Portal vein thrombosis
Chemoembolization (TACE)	Selective infusion of drug-eluting microparticles into hepatic artery or mixture of lipiodol and doxorubicin	Intermediate-stage HCC Unresectable intrahepatic cholangiocarcinoma Recurrent HCC	Child C Cirrhosis Terminal HCC Portal vein thrombosis
SIRT	Selective infusion of Y-90 containing microparticles into hepatic artery		Prior radiotherapy to region Perfusion to stomach or bowel on MAA study Prohibitive lung shunt on MAA study
Y-90 Radiosegmentectomy	Administration of high dose into 1–2 liver segments	Earlry-stage HCC not amenable to surgical resection or ablation Intermediate-stage HCC confined to 1 or 2 hepatic segments. Intrahepatic cholangiocarcinoma confined to 1 or 2 hepatic segments	Hepatic arterial anatomy precludes segmental administration of Y-90 microspheres
Lobar SIRT	Administration of lower dose to single lobe or staged treatment of entire liver.	Intermediate-stage HCC Advanced HCC Unresectable Intrahepatic Cholangiocarcinoma Recurrent HCC	Terminal HCC
Ablative Therapies		Early-stage HCC Unresectable intrahepatic cholangiocarcinoma Recurrent early-stage or intermediate-stage HCC	Irreversible coagulopathy Lack of safe percutaneous approach to tumor Peripheral or subcapsular tumor Hilar tumor
RFA	Thermal ablation with high-temperature probe		Tumor > 3 cm Unable to tolerate anesthesia or deep sedation
MWA	Thermal ablation with high-temperature probe		Tumor > 5 cm Unable to tolerate anesthesia or deep sedation
Cryoablation	Thermal ablation with low-temperature probe		Tumor > 5 cm
PEI	Chemical ablation with ethanol injection		Tumor > 3 cm

**Table 2:** Outcomes in locoregional therapy of hepatobiliary tumors.

Locoregional Therapy Outcomes		
Therapy	Hepatocellular Carcinoma	Intrahepatic Cholangiocarcinoma
Bland Embolization and TACE	Delayed progression in intermediate stage Possible increased survival in intermediate stage Possible delayed progression in advanced stage TACE possibly superior to bland embolization	Possible delayed progression
SIRT	Delayed progression in intermediate stage Possible increased survival in intermediate stage Possible imaging response in advanced stage	Possible delayed progression
Ablation	Equivalent survival to surgical resection in early-stage disease Possible delayed progression in intermediate stage	Possible delayed progression
